# ‘When helpers hurt’: women’s and midwives’ stories of obstetric violence in state health institutions, Colombo district, Sri Lanka

**DOI:** 10.1186/s12884-018-1869-z

**Published:** 2018-06-07

**Authors:** Dinusha Perera, Ragnhild Lund, Katarina Swahnberg, Berit Schei, Jennifer J. Infanti, on behalf of the ADVANCE study team, Elisabeth Darj, Mirjam Lukasse, Johan Håkon Bjørngaard, Sunil Kumar Joshi, Poonam Rishal, Rajendra Koju, Kunta Devi Pun, Kumudu Wijewardena, Munas M. Muzrif, Jacquelyn C. Campbell

**Affiliations:** 1 Department of Community Medicine, University of Sri Jayewardenepura, Colombo, Sri Lanka; 2 Department of Geography, Faculty of Social and Educational Sciences, Norwegian University of Science and Technology, Trondheim, Norway; 3 Department of Health and Caring Sciences, Faculty of Health and Life Sciences, Linnaeus University, Kalmar, Sweden; 4 Department of Public Health and Nursing, Faculty of Medicine and Health Sciences, Norwegian University of Science and Technology, Trondheim, Norway

**Keywords:** Obstetric violence, State health institutions, Intersectionality, Sri Lanka

## Abstract

**Background:**

The paper explores how age, social position or class, and linguistic and cultural background intersect and place women in varying positions of control and vulnerability to obstetric violence in state health institutions in Colombo district, Sri Lanka. Obstetric violence occurs during pregnancy, childbirth and the immediate postpartum period; hence, it is violence that directly affects women. The authors aim to break the traditional culture of silence around obstetric violence and bring attention to the resulting implications for quality of care and patient trust in obstetric care facilities or providers.

**Methods:**

Five focus group discussions were held with 28 public health midwives who had prior experience working in labor rooms. Six focus group discussions were held with 38 pregnant women with previous childbirth experience. Additionally, 10 of the 38 women, whom felt they had experienced excessive pain, fear, humiliation, and/or loss of dignity as patients in labor, participated in individual in-depth interviews. An intersectional framework was used to group the qualitative data into categories and themes for analysis.

**Results:**

Obstetric violence appears to intersect with systems of power and oppression linked to structural gender, social, linguistic and cultural inequities in Sri Lanka. In our dataset, younger women, poorer women, and women who did not speak Sinhala seemed to experience more obstetric violence than those with relevant social connections and better economic positions. The women in our study rarely reported obstetric violence to legal or institutional authorities, nor within their informal social support networks. Instead, they sought obstetric care, particularly for childbirth, in other state hospitals in subsequent pregnancies.

**Conclusions:**

The quality of obstetric care in Sri Lanka needs improvement. Amongst other initiatives, policies and practices are required to sensitize health providers about the existence of obstetric violence, and repercussions are required for abusive or discriminatory practices. The ethics of care should be further reinforced in the professional training of obstetric health providers.

## Background

No one wants to be humiliated or beaten by health staff [like I was], especially when we go for delivery. We expect love and care. We are helpless there. I think it is important to raise the staff’s awareness. I don’t know how to do that, but this would be the only way of changing them. I never wished to tell this story to anyone else, but today I told you everything because I want to help others to not have to face such unpleasant situations. (Excerpt from authors’ interview with a Tamil woman)

The fifth Millennium Development Goal motivated the development of health policies and reforms around the world aimed to increase the number of live births in health facilities and reduce maternal mortality. On a global level, maternal and infant health outcomes are improving, and maternal mortality ratios are declining. However, ensuring patient safety and high quality obstetric care is a lingering and unfinished agenda. There is growing evidence of a range of disrespectful and violent practices that women experience in obstetric care facilities at the hands of health care providers, particularly during childbirth [1, 2]. The literature reveals that mistreatment and violence can occur at the level of interaction between the woman and the provider, as well as through systemic failures at the health facility and health system levels [2, 3]. Furthermore, it includes intentional acts of emotional, verbal and sexual violence; a variety of obstetric practices that may inadvertently cause patient suffering, such as unnecessary episiotomies, abandonment or refusal to assist women during delivery; lack of provider empathy; and lack of consent for interventions such as caesarean section deliveries [1, 4–8]. Obstetric practices can also be perceived as mirroring the attitudes and practices of abusive men in intimate relationships through coercive control of women’s bodies and behaviors [9]. Consequently, obstetric care facilities can be poignant sites of violation and suffering for pregnant women and women in childbirth.

Although nearly all (98%) Sri Lankan women give birth in health facilities [10], the topic of their mistreatment or violence in obstetric care has not been investigated in the country. During our earlier research on the prevalence of domestic violence during pregnancy, and the role of public health midwives (PHMs) in providing antenatal (ANC) care to pregnant women living with domestic violence [11], we were confronted with stories from pregnant women who had experienced violence perpetrated by health care providers, especially during childbirth in state institutions. In addition, the guidelines for obstetric care in Sri Lanka instruct providers to screen pregnant women for domestic violence but these guidelines are not always followed. We therefore wanted to explore if there was an association between violence perpetrated by obstetric care providers and women’s trust in and willingness to reveal incidences of domestic and other forms of violence to these professionals.

Disrespectful and abusive treatment of women in labor may result from health system failures, including what is learned by health providers in training and reinforced on the job as well as various types of prejudice held in a society. In an attempt to identify some of the root causes of the problem in Sri Lanka, we engage with the theory of intersectionality in this paper [12–15]. Crenshaw’s [13] concept of intersectionality posits that social contexts are created by the intersections of systems of power (e.g. race, class, gender, and sexual orientation) and oppression (e.g. prejudice, class stratification, and gender inequality). Intersectionality colors the meaning and nature of violence, including how it is experienced by the self and responded to by others, and how it can be personal and/or imply social consequences in public spheres. Furthermore, in gender studies, where the concept of intersectionality is widely used, it is argued that gender entangles and intersects with other axes of social identity such as ethnicity, class, and age [14]. The theory of intersectionality postulates that insights into women’s everyday realities are best obtained when the factors linked to privilege, oppression, identity, positionality – and the situations in which women are placed – are explored simultaneously [14, 16]. Like Larson et al. [15], who build on Bowleg [17] and Hankivsky [18, 19], we find it instructive to use an intersectional framework in health systems research as

it allows us to improve our understanding of inequality [by] better reflecting the complexity of the real world. [Intersectionality] moves beyond understanding social hierarchies either in isolation from one another (e.g. gender as separate from race) or in an additive manner (e.g. gender plus race equals greater disadvantage). Instead, it highlights social categories (such as gender, age, class and race) as mutually constituted and intersecting in dynamic and interactive ways…intersectionality considers how individuals can simultaneously experience and embody privileges and disadvantage as different social hierarchies combine in various ways across time and diverse locations (p.2).

In this paper we explore how the axes of age, social position or class, and linguistic and cultural background intersect to place women in varying positions of control and vulnerability to obstetric violence in state health institutions in Colombo district, Sri Lanka. We draw on a duo of voices – those of PHMs, and those of women who felt they experienced excessive pain, fear, humiliation or loss of dignity as obstetric care patients. It is clear that the majority of health care providers in Sri Lanka, as around the world, are committed to providing appropriate patient care of a high ethical standard. However, the existence of transgressions of such care, as well as the relative invisibility of this aspect of violence against women, is a significant impediment to patient safety. Obstetric violence also undermines the quality of patient-provider relationships and patient trust in the health system.

Below we present the key concepts and contextual factors that anchor our study. Thereafter, we describe our research methodology; then we present our key findings on how PHMs and women who have given birth in state facilities either perceive of or have experienced violence during pregnancy and childbirth. This is followed by a discussion of how obstetric violence intersects with a patient’s age, social position, and linguistic and cultural background, and takes place within the health system and at individual and societal levels.

### Conceptualizing obstetric violence

Violence perpetrated by health care providers has been studied under the rubrics of different terms – for example, patient satisfaction [20], obstetric violence [21, 22], ethical transgressions by staff [23], and abuse in health care [24]. These studies provide ample scientific evidence that patients encounter violent or disrespectful experiences in many health care settings and types of patient-health provider relationships around the world. Knowledge about obstetric violence in the South Asia region is limited, however. Of the 65 studies included in Bohren et al.’s [2] systematic review on the mistreatment of women during childbirth in health facilities globally, only two were located in South Asia (India and Bangladesh) [25, 26]. Neglect, hurried support, and verbal abuse were considered the most rampant forms of violence reported in these studies. As the study of disrespect and violence during obstetric care develops worldwide it is important to explore the occurrence of such violence in Sri Lanka too.

We use the term ‘obstetric violence’ in this paper, building on other recent studies ([for example, 3, 27], to refer to mistreatment that occurs in the care provided during pregnancy, childbirth and the immediate postpartum period. Like Diaz-Tello et al. [3] we consider obstetric violence as “bullying and coercion of pregnant women during birth by health care personnel, [and]…a systemic problem of institutionalised gender-based violence” (p.1). Our work also supports Sadler et al.’s [27] argument that disrespect towards women during childbirth should be analysed as a consequence of structural violence, and that the term obstetric violence is a useful analytical tool for addressing structural violence in obstetric health care.

### Obstetric health care in Sri Lanka

Sri Lanka’s maternal health indicators are much lauded compared to those of many other low- and middle-income countries. The achievement of this success story is often credited to the country’s free-of-charge, decentralized system of curative, preventive and rehabilitative health services. The Ministry of Health’s Family Health Bureau is the dedicated focal point for maternal and child health (MCH) in the country, and MCH services exist at the community level across the country. Pregnant women are free to select MCH services in either the state or private sector, but all should be registered under the professional jurisdiction of a PHM. In 2013, 99% of pregnant women were visited at home at least once by a PHM [28]. In the same year, the percentage of institutional deliveries was 98% [10].

Normal (vaginal) deliveries occur in hospitals or maternity homes in Sri Lanka, which offer basic but sufficient obstetric care facilities. Caesarean sections are performed in hospitals that employ obstetricians. In all state facilities, pregnant women stay in antenatal wards until they are admitted to labor rooms and, following the birth, they stay in postnatal wards. State health institutions have no private rooms for women in labor, but each woman is provided with a separate delivery bed. Companions are not permitted to accompany birthing women in state hospitals.

Great advances have been made in the overall indices of health and quality of life for Sri Lankan women in recent decades, and the maternal mortality ratio (MMR) is currently significantly lower than in other South Asian countries. In 2013, the MMR in Sri Lanka was 29 per 100,000 live births, which is comparable to the USA, while the MMRs in regional neighbours, Nepal and India, were both 190 [29]. Nonetheless, disparities, discrimination and violations of women’s rights remain regular facts of daily life for Sri Lankan women. Much research has focused on the health and other consequences of violence against women in the country. Studies have explored violence experienced in intimate partner relationships [30–32], in occupational settings [33–35], in the estate sector [11, 36, 37], in special crisis situations [38], in public transport [39], and during childhood [40].

The magnitude of the problems, risk factors, and immediate and long-term implications of violence for the health and well-being of victims in the above-mentioned circumstances has been well-explored by various professionals. Possible strategies and interventions to overcome the pervasiveness of violence against women have also been identified, and some are being implemented at national or regional levels, particularly policies and laws. However, despite the increase in overall knowledge about violence against women, no research to date has documented violence experienced by women in health care facilities, although the issue has received attention in news media. A critical social dialogue regarding adverse events experienced by patients in health care in Sri Lanka emerged following an incident in which a 23-year-old woman was raped and murdered by a male doctor in a state hospital (*Sunday Times*, 3 September 2014, cited in [41]).

## Methods

We conducted the fieldwork for this study from May to July 2014, and in October 2015. Our team is an interdisciplinary collaboration of medical doctors, nurses and social scientists from Sri Lanka, Sweden and Norway. We were supported by two Tamil and Sinhala-speaking research assistants in the field, who acted as note-takers and translators. Ethical clearance was obtained from the Ethics Review Committee, Faculty of Medical Sciences, University of Sri Jayewardenepura in March 2014 (ref. no. 08/14).

The fieldwork took place in 10 ANC clinics in Colombo district. On average, an ANC clinic covers a population of 10,000. Colombo district was selected for its representativeness of Sri Lanka’s socio-economic, religious, and ethnic diversity. We invited PHMs working in the district to attend focus group discussions (FGDs), and pregnant women registered by the PHMs to attend separate FGDs. Later, we held follow-up in-depth individual interviews with ten of the pregnant women from the FGDs. Potential participants were contacted approximately one month in advance of the FGDs in person and given verbal information about the study aim. On the day of the group discussions, both with the midwives and pregnant women, written informed consent was obtained from willing participants after the study aim and objectives were explained again, and any questions had been clarified. We assured participants that their information was valued by us, and we shared the hope that the research could be useful for improving obstetric health services for other women in the country. The FGDs with both groups were carried out in private rooms at the ANC clinics or during non-clinic hours, in order to avoid disturbing routine activities. The data collection processes were guided by the World Health Organization’s recommendations for ethical and safe research on domestic violence against women [42]. We asked participants not to disclose identifying personal information, such as their names, during the interviews, and any of these types of details which made it into the audio-recordings were removed from the written transcripts of the interviews.

Five FGDs were held with a total of 28 PHMs who were involved in field-level MCH programmes in Colombo district at the time, and whom had prior experience working in state labor rooms. To ensure broad representation, the five groups comprised: PHMs working in urban environments, PHMs working in rural environments, junior PHMs (those with 3–4 years of work experience), senior PHMs (more than 15 years of work experience), and PHMs from the Tamil-speaking area of Colombo district. We consulted PHMs because one of their key roles in Sri Lanka is identifying, intervening, and referring pregnant women who experience violence [11]. The FGDs were conducted in Sinhala and translated into English by the first author.

Information was also gathered in 6 separate FGDs with a total of 38 pregnant women (6–7 women per group). These women were selected by the first author in consultation with the PHMs, and upon the women’s availability and willingness to participate. Six broad groups of pregnant women were consulted: young women (under 25 years old); middle-aged women (over 35 years); women living in urban areas; and rural areas; a well-educated group (educated at least up to secondary school level); and a Tamil-speaking group from the estate sector. Ten of these women – all whom felt they had previously experienced violence or disrespect during childbirth – were also invited to participate in follow-up, individual in-depth interviews. These ten women were selected by the first author, again in consultation with PHMs, to ensure diversity in terms of age, ethnicity, and urban or rural background. The FGDs and interviews with the pregnant women were conducted in Sinhala or Tamil; they were tape-recorded and notes were taken. The audio recordings were transcribed verbatim by the first author and then translated into English by her. In the process of translation, information was removed that could lead to identification of individual participants. A selection of the translated text was checked for language accuracy and consistency by a colleague who was not otherwise involved in the study.

Our study team has also carried out a large-scale quantitative survey assessing the prevalence of violence in health care in Colombo district (currently unpublished). This qualitative study was designed as part of the larger study to add greater depth to and scrutiny of knowledge obtained from the prevalence survey. The processes of data coding and analysis for this qualitative study were carried out collaboratively by the research team. We used an intersectional framework to group the data into categories (health system factors, individual factors, and sociocultural factors) and themes (age, social position, language and cultural background). Our analysis was also enriched by observations and visits to clinics and hospitals in Colombo district, as well as informal discussions with other health professionals such as Public Health Nursing Sisters (PHNS) and Medical Officers of Health (MOH).

## Results

### Views of public health midwives

In the past, PHMs were tasked primarily with delivering babies in homes or local clinics. Today though, with most deliveries taking place in health care institutions, PHMs provide a multitude of services in addition to maternal and neonatal care, such as family planning, immunizations, and growth and nutrition advice. The scope of midwifery in Sri Lanka has even extended to other public health activities, such as the control of communicable diseases and provision of care for victims of domestic violence. We consulted PHMs on whether or not they were aware of violence perpetrated by health care providers in MCH contexts. Nearly all participants agreed that such violence was prevalent: sanction

I will not try to safeguard my colleagues. Yes, it is happening…and not only in the hospital…Also, some of us in the field are responsible for certain occurrences of abuse.

Some of the PHMs felt obstetric violence was common, and some had heard stories from their relatives who, as patients, had experienced what they regarded as violence.

One midwife shared an exemplifying account of how she came to acquire second-hand knowledge of violence perpetrated by one of her colleagues. She explained that a mother of one child had asked her about the possibility of obtaining a ‘permanent family planning method’ (sterilization):

I asked the woman for the reason why she wanted a permanent method…At last she came out with the true story...A terrible story to tell…[She] had been hit in the hospital...She was suffering a lot from the incident. She didn’t want any more children because of what had happened.

Despite the shared perception in the FGDs that obstetric violence was common, it appeared that the majority of PHMs viewed this phenomenon as relatively trivial. The PHMs explained that giving clear and ‘firm’ instructions about how birthing women ‘should behave’ during delivery was part of the role of ‘a good midwife’. Few of them had reflected on how patients might perceive ‘firm’ behavior as unfair, humiliating, disempowering or even as acts of violence.

The PHMs also held the view that pregnant women were differently susceptible to obstetric violence. They identified the patients most at risk as follows:

those who scream unnecessarily;

those who are not ready to tolerate pain;

very young patients who do not comply with commands to strain and push during labor;

experienced patients [who] behave like they know everything;

[women who ask] unnecessary questions.

The PHMs mentioned many incidences of unwanted pregnancies, particularly among women returning home after working abroad, teenagers, and commercial sex workers. They expressed a genuine concern for such women and their babies. They told us it was the responsibility of both field-based and hospital midwives to ensure the safety of these women, and that this could require stern and firm behaviour. Moreover, the PHMs remarked that some women may not know when they are in a potentially risky situation during pregnancy or childbirth. They also mentioned that some women arrived at hospitals when birth was imminent but were ‘unprepared’, having not brought the clothing or other personal items required of them in state facilities. Furthermore, most PHMs felt that clinic and hospital environments created the underlying conditions for the potential mistreatment of patients. They highlighted poor infrastructure and congestion, in addition to lack of staff and heavy workloads, as factors contributing to their stressful professional realities.

### Women’s accounts of obstetric violence

Our FGDs with pregnant women who had previously delivered babies in state health institutions revealed many experiences of obstetric violence, particularly in labor rooms. By and large, the women in our study had kept silent about such misconduct. They were not aware of formal means for reporting the unacceptable behaviour of health care providers, and they believed that complaining (even informally) could result in potential harm to their babies. Two or three women in each FGD had personal adverse experiences with health care providers during childbirth, and several knew of close friends, siblings or relatives who had similar negative experiences. Still, the women expressed gratitude towards the health care system in Sri Lanka as a whole, and to individual health care providers for helping them to give birth safely. They did not want to challenge the system nor the individual care providers whom they depended on for continuing and future care. Additionally, regardless of their negative experiences, the women considered the safe births of their children to be of most importance, and they often justified the violence they had experienced as their *karma* (fate) in life. Nevertheless, mistreatment and abusive experiences led women to mistrust obstetric health facilities and providers. Upon experiencing violence or hearing of other women’s negative experiences at certain hospitals, the women strategized to seek care from different hospitals in subsequent pregnancies.

#### Verbal, emotional and sexual violence

Most of the women in the FGDs described their most poignant experiences of verbal or emotional violence perpetrated by obstetric care providers during the period of childbirth. Many women identified feeling ‘very upset’, ‘insulted’, ‘embarrassed’, ‘stupid’, ‘shocked’, and/or ‘bewildered’ while in labor:

All the time, she [the nurse] was blaming me, telling me that I was a headache to her. As I understand, I didn’t do anything wrong.

The women differed in the ways they reacted to these experiences but, in general, they seldom informed anyone about them, including their informal support networks of friends and family:

We very rarely say anything about these things to others, or we do not complain about these things to anyone [at all].

Although we experience things, we keep quiet. We do not argue back.

I bore it [the violence]. It was my fate.

The follow-up individual interviews gave further insights into the types of obstetric violence that occurred, which included sexual violence. One woman informed us about being sexually violated by a male hospital employee in the operating theatre during her first delivery. Her baby was delivered by caesarean section and after the surgery she was lying on a trolley in the theatre waiting to be taken to the postnatal ward. The nurses were busy and no one was attending to her. She fell asleep and awoke due to a feeling she described as ‘unusual touching’. Upon opening her eyes, she found a male employee standing over her:

I was shocked. One of his hands was on my breast.

This woman described immediately calling loudly for a nurse, which caused the man to run out of the theatre. However, she did not complain further to anyone about the incident because she felt too embarrassed to talk about what had happened.

#### Violence, class, and social position

The women in our study held the perception that they were treated differentially in labor in state hospitals depending on their financial means, linguistic and cultural backgrounds, and social status. One woman explained that personal connections mattered greatly to the quality of obstetric care received. For example, being a relative of a health care provider, or a relative of someone known to a health care provider, was sufficient to prevent the occurrence of undesirable behaviors:

I have seen enough staff blame mothers. I had no problem though because my sister worked in the same hospital, but I am not afraid to say that some of them [health care providers] are very rude to their patients.

Another study participant’s experience illustrates how economic and social status can play a role in the experience of violence during childbirth. She had limited formal education and socioeconomic means, and no family support during the pregnancy. However, she felt well-supported by the midwife at her regional ANC clinic and had received some money from other pregnant women at the ANC clinic to buy food and other essentials for the baby. She described feeling emotionally and physically violated by the labor room nurse and midwife:

To start, when I went to the labor room they were not happy about my clothes. They were too big for me [because] I was wearing what I was given by a friendly woman.

While in labor she passed a stool on the bed and was asked to provide another bed cloth but she only had one and did not want to spare it yet. This resulted in the midwife throwing a bed sheet at her and telling her she was ‘like a toilet’. The nurse who attended to her just before the birth also blamed the woman for ‘messing up’ the labor room:

She cursed me, telling me that even though I had not a cent to buy a cloth I had got ‘the other things’ [that is, becoming pregnant] done ‘in good time’.

During the final stages of delivery, when the woman was in severe pain, she accidentally touched a midwife who was standing near her. The midwife’s response was to turn and slap the woman over her hands, yelling at her not to touch her.

The woman’s attitude towards the labor room doctors differed however from her attitudes towards the midwife and nurse. She felt she had been treated well by the doctors and had not experienced any discrimination while under their care. She deplored the unavailability of doctors in the labor room throughout the birth:

Labor room nurses and midwives blame and hit. Doctors usually do not. The others only behave badly when the doctors are out of the labor room. When the doctors are in the room, they behave differently. Especially when a VOG [consultant obstetrician] is there, it is totally different. They [the nurses and midwives] are much friendlier then and appear kind. I think they want to show their bosses that they are human.

The woman did not make any formal complaints about her care, and explained to us, ‘We are poor people. Where else can we go [for childbirth]?’

#### Violence and teenage pregnancy

Being pregnant at a young age appeared to significantly influence whether women experienced violence from obstetric care providers. A mother whom we met in her second pregnancy described her first birth experience to us, which was two years prior to our interviews, when she was not yet an adult. The woman had left school early and married at a young age. She was unaware of family planning methods at the time of their marriage and became pregnant shortly thereafter. She regularly visited the local ANC clinic and hospital during her pregnancy, but was not informed about the pain or processes of childbirth. She delivered her baby after many hours of severe labor pain:

I still feel so upset to be reminded about what happened. That second ‘Sir’ [doctor] came to me and from the very first moment stared at me and asked me in a rude way to keep my legs in ‘the correct position’ [for him] to check [the progress of the labor]. I did as he asked. Oh god! How terrible! That was the moment I felt the most severe pain during the entire labor – when he was checking me. I had no control and screamed loudly.

When she was crying with pain the doctor scolded her. Later in the labor, she was asked to push when the contractions came, but she felt too exhausted after many hours of pain:

Then that doctor came close to me and pinched me on my shoulder, asking [me] to push, but I was weak. Then he slapped me on my thighs vigorously. Other staff around him kept silent.

The woman felt she had no recourse during the incident and did not inform anyone about it. She was not aware of any repercussions for the unacceptable behavior on the part of her care providers nor whether any measures existed for reporting it, such as to the police or health authorities. Her inability to protest her perceived mistreatment was typical of many other women’s experiences described to us during our data collection.

#### Violence, language and cultural background

Tamil and Muslim women in our study believed that prejudice related to their language or cultural practices was an underlying factor in mistreatment experienced during obstetric care, particularly verbal and emotional violence during childbirth. They described experiences when Sinhalese hospital staff insulted them, as illustrated in the following quote where a Sinhalese hospital nurse is speaking to a Muslim woman:

You are the people who bring headaches to us. We are the people who always face trouble because of you…You will produce children year by year starting from 19 [years], but we have to resolve all your problems.

Tamil-speaking women in our interviews stressed the importance of reducing language barriers in state health institutions. Although most of the important documents related to antenatal care and childbirth are written in the two main languages in Sri Lanka, the Tamil women in our study explained that hospitals still often lack simple information in their main language – for example, ‘lists of items to bring for childbirth’ and ‘essential commands to know during labor’.

One study participant, a well-educated Tamil woman, described experiencing verbal violence and disrespect by a midwife in the antenatal ward of a state hospital in a prior pregnancy. According to her, although she was unable to read Sinhala fluently (her second language), she could understand and speak it. When she had asked the midwife if she could have a Tamil copy of the list of instructions to prepare for childbirth to check whether she had understood everything in the Sinhala version of the list, the midwife yelled at her:

The most fitting term for how she [the midwife] spoke to me is that she ‘barked’ at me. She asked me very impolitely whether I had come to make changes in the hospital, and she was cursing at me, telling me that they had already done enough for Tamils.

Our participant said she had argued with the midwife because she did not see any reason why she should be insulted or blamed. She felt that discrimination from health care providers relating to culture and language was unacceptable:

We respect and owe these people, but some changes in their attitudes are needed.

A Muslim woman, well-educated and married to a man in secure employment, explained experiencing discrimination in both the local ANC clinic and the hospital due to her language and culture:

During my first pregnancy, I was living in an area where there were no Muslims at all. Here [referring to current pregnancy], the majority of the villagers are Muslims, so the doctors and other health workers are used to our traditions and culture...[But in the previous location] no one bothered to educate me or, at the least, speak with me. I was so worried at times when they were calling me ‘the Muslim mother’. They were calling the other women by their names. I would have also liked it if they called me by my name.

When she was admitted to the antenatal ward of a state hospital for the birth of her first child she was immediately scolded by a nurse who had asked her to change her clothes:

I went to the ward in a *shalwar*. That nurse looked at me from top to toe. In front of the other women, she scolded me for my dress and asked me to go put on a cloth and a bed jacket. She laughed at me telling me that I couldn’t give birth to a child covered in clothes from head to toe.

The woman had never told the health providers in her local clinic that she felt upset and insulted in the hospital, and claimed the underlying reason for her lack of disclosure was a personal lack of courage.

## Discussion

### Intersecting dimensions of violence

Discussions about intersectionality and violence against women inform the phenomenon of obstetric violence [43], where various axes of power and oppression operate in mutually constituted and interactive ways to place women in different positions of vulnerability to mistreatment or violence. Intersectionality suggests that no one dimension, such as gender inequality, can be privileged as explanatory of gender-based violence on its own. Rather, gender inequality is in itself modified by its intersection with other systems of power and oppression [12]. In the results we have presented, the prevailing systems of power and oppression in obstetric care stem from the intersection of various health system, individual and socio-cultural factors. We discuss these factors separately in turn in the remainder of this section. Then, in our conclusions section, we draw our argument together by demonstrating the intersecting ways in which experiences of obstetric violence are shaped.

### Health system factors

In the larger literature on abuse in health care, social and institutional norms that are accepting of violence against women play a central role [27]. This includes women’s experiences of and reactions to their (mis-)treatment, and providers’ relative lack of emotional empathy or understanding for their patient’s perceptions of mistreatment [44]. We found that women in our study typically remained silent about their experiences because they accepted disrespect and violence as ‘normal’ in health care settings, and were largely unaware of their rights as patients to respectful treatment and care in health facilities [45, 46].Women’s silence about experiences of obstetric violence also represents an imbalance in power dynamics between patients and medical staff who appear to be able to use controlling verbal and physical behaviors without repercussion. However, Jewkes and Penn-Kekansa [47] urge us to avoid ‘blaming the health workers as a group’ . The stressful and otherwise poor working environments for many obstetric providers, with hospital overcrowding and shortages of staff, contribute to demoralization. Sadler et al. (2016) suggest that such health system factors should in themselves “be framed as forms of disrespect and abuse, as [should] the consequences of being socialised within – and driven to exercise – violence” ([27], p. 51).

As our study indicates, violence in obstetric care is also related to a clash of perceptions between patients and providers – for example, patients perceive being told how to behave during labor as a form of violence, while providers consider that giving such ‘moral instruction’ is part of the role of effective nursing or midwifery. Additionally, midwives’ perceptions of patients’ non-compliance with their advice may be genuinely rooted in safety concerns for the women (e.g. because women may not know when they are in a risky situation).

The above-described health system factors need to be considered in future policy discussions to improve obstetric care in Sri Lanka, particularly when raising questions about who should be holding health care providers accountable to their patients, and how. Addressing the current status of limited accountability for unacceptable behaviour in care relationships should be a matter of state responsibility. Better supervision, training, and policies to ensure ethical conduct and patient safety must be formulated and enforced in obstetric care. Cultural ideas regarding who ‘deserves’ health care, who should provide such care, and what kind of obstetric health system is desired for the country, need to be debated and reconsidered as these are the bases for fairer, more gender-sensitive, and appropriate care practices [48].

### Individual factors

In our study, a woman’s *age* influenced her experience of obstetric violence. Teenage mothers appeared particularly vulnerable to obstetric violence, evidently due to their inexperience with the health system and lack of knowledge about what childbirth entails. Research from other countries, such as Kenya, has yielded similar findings [49].

Women’s *poor economic status* was also identified as a contributing factor to obstetric violence. Women who can afford to give birth in private hospitals are typically assured better privacy and user-friendly care as they can bring companions into separate delivery rooms as potential advocates (or at least witnesses). For the majority of women in Sri Lanka, however, who give birth in state institutions, there is no choice but to acquiesce power and entrust their health exclusively to their obstetric care providers. Where mistrust arises in this patient-provider relationship, it appears the only recourse or option that women find practical and acceptable is to identify a different state hospital with a better reputation in subsequent pregnancies.

Low socio-economic status has been identified as increasing the risk of obstetric violence in other countries as well. In Burkina Faso, for example, poor and rural women avoid visiting health care facilities during pregnancy because they are treated in an abusive manner there [50]. A study conducted among women in South Africa reiterated that women with low status in terms of economy, and women who are less educated, are more prone to abuse in health care because the providers know the women will accept whatever standard of care they receive, even if it is minimal [51].

### Socio-cultural factors

Three socio-cultural factors seemed to play an important role in women’s experiences of obstetric care in our study: patriarchy, patient-provider hierarchies, and the linguistic and cultural backgrounds of patients. These factors are intertwined and may explain the lack of attention thus far given to obstetric violence in the country. For example, women’s passive acceptance of mistreatment and violence in obstetric care, and their inability to voice it even within their informal support networks, may be linked to the power structures and socio-cultural expectations underlying both patriarchy and patient-provider relationships. In other words, women’s general submissiveness to men in patriarchal societies like Sri Lanka may also influence their perceptions of and social norms about their interactions with other people considered their ‘superiors’, such as health care providers, even where the provider is also female. Hence, the gender inequity that underlies obstetric violence is embedded in the dynamic interaction of patriarchy and patient-provider hierarchies.

Furthermore, although Sri Lanka is well known for its good and decentralized health care system, there is increasing polarity between the state and private systems which is leading to another hierarchy of patients and providers in the country, especially in urban areas of the country. We learned that urban-dwelling Muslim women with the necessary financial means often opt for obstetric care in private institutions where they feel privacy and language concerns are fewer. Hence, here, patient hierarchies intersect with their language and culture.

Studies from other countries document that obstetric violence relates to women’s cultural backgrounds (for example, see [52] on indigenous women in Peru). In the USA, race is a known contributing factor in emotional abuse and discrimination [53]. In our interviews too, Tamil and Muslim women, particularly those who did not speak or understand Sinhala, felt their language and cultural practices were risk factors for potential mistreatment in obstetric care settings in Colombo district. As with all qualitative research, we must be cautious in assigning potential associations here as our sample is neither large nor diverse enough for the results to be generalized on a national level. Further research is required to explore how minority language, cultural and ethnic status may impact the perception or experience of differential treatment in obstetric care settings in Sri Lanka.

## Conclusions

We have documented that pregnant women encounter obstetric violence in Sri Lanka and, by and large, do not report or complain about it even within their informal networks. PHMs explain mistreatment and violence in the provision of obstetric care as a result of women not following medical advice or being too assertive or otherwise uncooperative, unprepared or ill-informed. PHMs also attributed the occurrence of obstetric violence to the lack of resources at the institutional level. In general, the women in our interviews identified the perpetrators of obstetric violence among junior doctors, nurses, and midwives. At the same time, though, they appreciated the care and actions of some health providers from these same professions whom they had witnessed holding their colleagues to account for creating discriminative or abusive environments. Hence, the identities and practices of the women and health care providers intersect as well to shape perceptions and experiences of obstetric violence.

In our study population, younger pregnant women appeared to suffer more violence in obstetric care than older women; Tamil and Muslim women who could not speak Sinhala suffered more violence than Sinhalese women; and poorer women experienced more violence than those with relevant social connections or better economic positions. In general, women explained that they felt disempowered during childbirth, again particularly women who were young, inexperienced with the health care system, and had little support from family or personal connections to health staff. Also, some particular groups of women, such as sexually active teenagers, seemed to be regularly singled out in the health system for being unprepared or uncooperative in childbirth. Thus, some health care providers appeared to being making judgements about the relative ‘worth’ of patients, with implications for the quality of care provided.

As stated earlier, one of our original aims for this study was to investigate a potential link between the mistreatment of women in obstetric care and their relative willingness to reveal domestic and other forms of violence to health care providers. Our data is inconclusive in this respect. However, it does point to an absence of recourse for patients who experience mistreatment in obstetric care settings and a lack of repercussions or accountability for abusive care providers. A forthcoming paper from our study team will explore the prevalence of abuse experienced by women in the state health care sector in Sri Lanka and associated factors, including the characteristics of women who have disclosed gender-based violence to health care providers. We suggest that future research situates the mistreatment of women during childbirth in a broader framework of gender-based violence to explore potentially overlooked connections. Also, the women in our study were happy to be given a forum to voice their experiences of obstetric violence; thus, we suggest that opportunities be provided for patients to comment on the quality of care received as standard practice, perhaps in the form of exit interviews from hospitals.

Making obstetric violence visible is a first step for corrective actions to improve the emotional and physical safety and overall quality of care for patients, and to ensure dialogue between patients and health providers is based in mutual respect, trust and understanding. It follows that obstetric violence has to be dealt with as a structural problem articulated at individual, health system, and socio-cultural levels. Sadler et al. [27] outline a host of initiatives to address structural violence in childbirth settings specifically – at legislative, economic, organizational, educational and research levels. Below, briefly, we build on their suggestions that are relevant for our present and future work.

We propose health system reforms and improvements in Sri Lanka to ensure professional accountability for the safety and wellbeing of patients, and formal measures for patient recourse for cases of serious violence, such as the provision of patient advocates and impartial medical boards to receive and review complaints. In addition, the ethics of care should be further reinforced in the professional training of all obstetric health providers. Regular refresher training should be offered and possibly mandated; and there could be an increased focus in the training on cultivating provider’s empathy for patients through experiential learning and techniques such as theatre [7]. Finally, state hospitals could introduce standardized measures to capture patients’ perspectives on quality of care. Relatedly, the government could consider introducing incentives for hospitals to improve their quality of care.

## Abbreviations

ANC: Antenatal care
FGD: Focus group discussion
MCH: Maternal and child health
MMR: Maternal mortality ratio
PHM: Public health midwife

## References

[1] d’Oliveira AF, Diniz SG, Schraiber LB (2002). Violence against women in health-care institutions: an emerging problem. Lancet.

[2] Bohren MA, Vogel JP, Hunter EC, Lutsiv O, Makh SK, Souza JP, Aguiar C, Saraiva Coneglian F, Diniz ALA, Tunçalp Ö, Javadi D, Oladapo OT, Khosla R, Hindin MJ, Gülmezoglu AM. The mistreatment of women during childbirth in health facilities globally: a mixed-methods systematic review. PLoS Med. 2015;12(6) 10.1371/journal.pmed.1001847. 10.1371/journal.pmed.1001847 PMC4488322 26126110

[3] Diaz-Tello F (2016). Invisible wounds: obstetric violence in the United States. Reprod Health Matters.

[4] D’Ambruoso L, Abbey M, Hussein J (2005). Please understand when I cry out in pain: women’s accounts of maternity services during labour and delivery in Ghana. BMC Public Health.

[5] Jewkes R, Abrahams N, Mvo Z (1998). Why do nurses abuse patients? Reflections from south African obstetric services. Soc Sci Med.

[6] Smith-Oka V (2013). Managing labor and delivery among impoverished populations in Mexico: cervical examinations as bureaucratic practice. Am Anthropol.

[7] Shapiro J (2008). Walking a mile in their patients’ shoes: empathy and othering in medical students’ education. Philos Ethics Humanit Med.

[8] Kruk ME, Kujawski S, Mbaruku G, Ramsey K, Moyo W, Freedman LP. Disrespectful and abusive treatment during facility delivery in Tanzania: a facility and community survey. Health Policy Plan. 2014; 10.1093/heapol/czu079. 10.1093/heapol/czu079 29304252

[9] Charles S (2011). Obstetricians and violence against women. Am J Bioeth.

[10] Senanayake H, Goonewardene M, Ranatunga A, Hattotuwa R, Amarasekera S, Amarasinghe I (2011). Achieving millennium development goals 4 and 5 in Sri Lanka. BJOG.

[11] Infanti JJ, Lund R, Muzrif MM, Schei B, Wijewardena K (2015). Addressing domestic violence through antenatal care in Sri Lanka’s plantation estates: contributions of public health midwives. Soc Sci Med.

[12] Bograd M (1999). Strengthening domestic violence theories: intersections of race, class, sexual orientation, and gender. J Marital Fam Ther.

[13] Crenshaw K, Fineman M, Mykitiuk R (1994). Mapping the margins: intersectionality, identity politics and violence against women of color. The public nature of private violence.

[14] Lund R, Doneys P, Resurrección BP (2015). Gendered entanglements: revisiting gender in rapidly changing Asia.

[15] Larson E, George A, Morgan R, Poteat T. 10 best resources on… intersectionality with an emphasis on low- and middle-income countries. Health Policy Plan. 2016; 10.1093/heapol/czw020. 10.1093/heapol/czw020 27122486

[16] Coles A, Gray L, Momsen J (2015). The Routledge handbook of Gender Development.

[17] Bowleg L (2012). The problem with the phrase women and minorities: intersectionality–an important theoretical framework for public health. Am J Public Health.

[18] Hankivsky O (2012). Women’s health, men’s health, and gender and health: implications of intersectionality. Soc Sci Med (1982).

[19] Hankivsky O (2014). Intersectionality 101.

[20] Coyle J (1999). Understanding dissatisfied users: developing a framework for comprehending criticisms of health care work. J Adv Nurs.

[21] Perez D’Gregorio R (2010). Obstetric violence: a new legal term introduced in Venezuela. Int J Gynaecol Obstet.

[22] Sánchez SB (2014). Obstetric violence: medicalization, authority abuse and sexism within Spanish obstetric assistance - a new name for old issues? (dissertation for MA degree in women’s and gender studies).

[23] Brüggemann AJ, Wijma B, Swahnberg K (2012). Abuse in health care: a concept analysis. Scand J Caring Sci.

[24] Swahnberg IM, Wijma B (2003). The NorVold abuse questionnaire (NorAQ): validation of new measures of emotional, physical, and sexual abuse, and abuse in the health care system among women. Eur J Pub Health.

[25] Hulton LA, Matthews Z, Stones RW (2007). Applying a framework for assessing the quality of maternal health services in urban India. Soc Sci Med.

[26] Afsana K, Rashid SF (2001). The challenges of meeting rural Bangladeshi women’s needs in delivery care. Reprod Health Matters.

[27] Sadler M, Santos MJDS, Ruiz-Berdún D, Rojas GL, Skoko E, Gillen P, Clausen JA (2016). Moving beyond disrespect and abuse: addressing the structural dimensions of obstetric violence. Reprod Health Matters.

[28] Family Health Bureau (2013). Annual report on family health.

[29] World Bank’s world development indicators 2015. http://documents.worldbank.org/curated/en/2015/04/24352249/world-development-indicators-2015. Accessed 21 Apr 2016.

[30] Deraniyagala S (1992). An investigation into the incidence and causes of domestic violence in Colombo, Sri Lanka.

[31] Jayasuriya V, Wijewardena K, Axemo P (2011). Intimate partner violence of women in the capital province of Sri Lanka: prevalence, risk factors and help seeking. Violence Against Women.

[32] Nirthanan P (1999). The extent and some factors associated with wife beating in the MOH area Kantale (dissertation for the degree of Doctor of Medicine in Community Medicine).

[33] Adikaram AS (2005). Is sexual harassment a problem in Sri Lankan workplaces?.

[34] Attanapola CT (2004). Changing gender roles and health impacts among female workers in export-processing industries in Sri Lanka. Soc Sci Med.

[35] Gamage PG (1999). Sexual harassment at the workplace: policy survey report.

[36] Palaniappan G (2003). Study on violence against women in the Plantations Communities Project (PCP) Estates.

[37] Wijayathilake K (2003). Harsh realities: a pilot study on gender based violence in the plantations sector. Colombo: plantation human development trust.

[38] Wijayathilake K (2004). Study on sexual and gender based violence in selected locations in Sri Lanka.

[39] Amarasinghe MP, Appuhamy HDSCP, Arandara DC, Perera J. A study of sexual harassment of women using public transport. In: Center for Women’s Research, editor. Gender, society and change. Colombo: Center for Women’s Research; 2005. p. 88-110.

[40] Perera B, Ostbye T, Ariyananda PL, Lelwala E (2009). Prevalence and correlates of physical and emotional abuse among late adolescents. Ceylon Med J.

[41] PKDC P (2015). Prevalence and correlates of adverse experiences in health care among antenatal women in the district of Colombo, Sri Lanka (dissertation for the degree of Doctor of Medicine in Community Medicine).

[42] World Health Organization (2001). Putting women first: ethical and safety recommendations for research on domestic violence against women.

[43] Hankivsky O, Reid C, Cormier R, Varcoe C, Clark N, Benoit C, Brotman S (2010). Exploring the promises of intersectionality for advancing women’s health research. Int J Equity Health.

[44] Freedman LP, Ramsey K, Abuya T, Bellows B, Ndwiga C, Warren CE, Kujawski S, Moyo W, Kruk ME, Mbaruku G (2014). Defining disrespect and abuse of women in childbirth: a research, policy and rights agenda. Bull World Health Org.

[45] Brüggemann AJ, Swahnberg K (2013). What contributes to abuse in health care? A grounded theory of female patients’ stories. Int J Nurs Stud.

[46] Bowser D, Hill K (2010). Exploring evidence for disrespect and abuse in facility-based childbirth: Report of a landscape analysis.

[47] Jewkes R, Penn-Kekana L. Mistreatment of women in childbirth: time for action on this important dimension of violence against women. PLoS Med. 2015;12(6) 10.1371/journal.pmed.1001849. 10.1371/journal.pmed.1001849 PMC4488340 26126175

[48] Moral Economies of Medicine Working Group. http://medicalanthropology.unc.edu/working-groups/. Accessed 22 Dec 2016.

[49] Center for Reproductive Rights & Federation of Women Lawyers-Kenya (FIDA) (2007). Failure to deliver: violations of women’s human rights in Kenyan health facilities.

[50] Amnesty International (2009). Giving life, risking death: maternal mortality in Burkina Faso.

[51] Fonn S, Xaba M (2001). Health workers for change: developing the initiative. Health Policy Plan.

[52] Kayongo M, Esquiche E, Luna MR, Frias G, Vega-Centeno L, Bailey P (2006). Strengthening emergency obstetric care in Ayacucho, Peru. Int J Gynaecol Obstet.

[53] Esposito NW (1999). Marginalized women’s comparisons of their hospital and freestanding birth center experiences: a contrast of inner-city birthing systems. Health Care Women Int.

